# Volatile Profiling of *Magnolia champaca* Accessions by Gas Chromatography Mass Spectrometry Coupled with Chemometrics

**DOI:** 10.3390/molecules27217302

**Published:** 2022-10-27

**Authors:** Chiranjibi Sahoo, Bibhuti Bhusan Champati, Biswabhusan Dash, Sudipta Jena, Asit Ray, Pratap Chandra Panda, Sanghamitra Nayak, Ambika Sahoo

**Affiliations:** Centre for Biotechnology, School of Pharmaceutical Sciences, Siksha ‘O’ Anusandhan (Deemed to Be University), Bhubaneswar 751003, Odisha, India

**Keywords:** *Magnolia champaca*, *Michelia champaca*, essential oil, chemometrics analysis

## Abstract

*Magnolia champaca* (L.) Baill. ex Pierre of family Magnoliaceae, is a perennial tree with aromatic, ethnobotanical, and medicinal uses. The *M. champaca* leaf is reported to have a myriad of therapeutic activities, however, there are limited reports available on the chemical composition of the leaf essential oil of *M. champaca*. The present study explored the variation in the yield and chemical composition of leaf essential oil isolated from 52 accessions of *M. champaca*. Through hydrodistillation, essential oil yield was obtained, varied in the range of 0.06 ± 0.003% and 0.31 ± 0.015% (*v*/*w*) on a fresh weight basis. GC-MS analysis identified a total of 65 phytoconstituents accounting for 90.23 to 98.90% of the total oil. Sesquiterpene hydrocarbons (52.83 to 65.63%) constituted the major fraction followed by sesquiterpene alcohols (14.71 to 22.45%). The essential oils were found to be rich in β-elemene (6.64 to 38.80%), γ-muurolene (4.63 to 22.50%), and β-caryophyllene (1.10 to 20.74%). Chemometrics analyses such as PCA, PLS-DA, sPLS-DA, and cluster analyses such as hierarchical clustering, i.e., dendrogram and partitional clustering, i.e., K-means classified the essential oils of *M. champaca* populations into three different chemotypes: chemotype I (β-elemene), chemotype II (γ-muurolene) and chemotype III (β-caryophyllene). The chemical polymorphism analyzed in the studied populations would facilitate the selection of chemotypes with specific compounds. The chemotypes identified in the *M. champaca* populations could be developed as promising bio-resources for conservation and pharmaceutical application and further improvement of the taxa.

## 1. Introduction

Essential oils are volatile organic compounds with a strong aroma. These are produced by aromatic plants as secondary metabolites to protect against microbes, fungi, viruses, and insects. The essential oils attract pollinators to disperse pollens and seeds and also repel insignificant others [1]. Essential oils (EOs) possess various bioactivities, i.e., antimicrobial, anti-inflammatory, antioxidant, etc., which make them a valuable commercial product in the pharmaceutical, cosmetic, food, and beverages industries [2,3]. As evidenced by literature survey, there is growing research interest in essential oils of various aromatic plants and their bioactive components [4,5,6]. An essential oil’s biological activity might result from the presence of only one active ingredient or the synergistic interactions of numerous different molecules [5]. Numerous researches have demonstrated the connection between essential oil variations and their bioactivities [2,7]. The yield and quality of essential oils are greatly influenced by genetic makeup, agronomic practices, plant age, climate, soil type, and composition [8,9,10]. Edaphic and abiotic factors affect the biosynthetic pathways of the volatile compounds, thereby leading to the development of chemotypes in a single species [11]. Furthermore, understanding the diversity of EOs would also make it easier to market certain chemotypes for use in food or phytopharmaceutical utilizations as well as for breeding better genotypes [3,12]. Therefore, a qualitative and quantitative analysis of EOs collected from different geographical regions would necessitate developing a promising bioresource for industrial purposes.

*Magnolia champaca* (L.) Baill. ex Pierre (syn. *Michelia champaca* L.), commonly known as Champak/Swarna Champa/Golden Champa, is a tall and magnificent evergreen tree belonging to the family Magnoliaceae. It is well known for its soothing and beautiful aromatic flowers, which help in relaxation. Geographically, *Magnolia champaca* is distributed throughout tropical and sub-tropical regions of Asia covering India, Nepal, China, Indonesia, Myanmar, Vietnam, Sri Lanka, Malaysia, and Thailand [13]. In India, wild populations of Champak are mainly found in the sub-Himalayan zone, south India, western ghat, and Assam. Traditionally, several parts of the plant are utilized in the treatment of various diseases such as inflammation, eye disorders, leprosy, cephalalgia, cough, gout, fever, colic, and antidote for scorpion and snake venoms, etc. [14]. The plant is reported to have several substantial pharmacological activities such as anti-diuretic, memory-enhancing, anti-diabetic, anti-inflammatory, antioxidant, antitumor, anti-microbial, and wound-healing properties [13,14].

*M. champaca* leaves are simple, alternate, and spiral, with a lamina of about 10–25 cm, lanceolate to elliptic-lanceolate, acuminate apex, acute base, the margin is slightly undulate, glabrous, strongly and reticulately nerved [15]. The leaf extract of *M. champaca* possesses anti-fertility, antibacterial, anti-inflammatory, antioxidant, analgesic, cytotoxic, antiulcerogenic, pro-cognitive, and helmintholytic activities [14,16,17,18,19,20,21]. Additionally, the leaves in an acidic medium inhibit mild steel corrosion [22]. Further, Champak leaf juice mixed with coconut oil is used for hair cleaning and removing lice and dandruff [23]. In addition, the market value of the dry leaf powder is $50 (for 100 leaves) and the essential oil is $13 (for 100 mL) (Sajee Sales; Shiva exports, India).

In spite of having enormous potential, few reports are available on phytoconstituent analysis of the leaf essential oil of *M. champaca* [24,25]. The available reports show that the *M. champaca* leaf essential oil is rich in sesquiterpenoids such as β-elemene, β-caryophyllene, α-humulene, β-selinene, and α-selinene, etc. [25]. However, to date, there is no report available regarding the variations in yield and phytochemical content of *M. champaca* leaf essential oil, necessitating the comparative assessment of essential oil among different populations.

Thus, the present research was conducted to explore the intraspecific variations in the phytochemical composition of *M. champaca* leaf essential oil collected from different geographic regions of Odisha, India. In order to find out the relationship among the accessions of *Magnolia champaca*, different chemometric analyses such as agglomerative hierarchical clustering (HCA), principal component analysis (PCA), partial least squares projection of latent structures (PLS), and partial least squares regression discriminant analysis (PLS-DA) were performed based on the essential oil compositions of the accessions. The results thus obtained were used to determine the *Magnolia champaca* chemotypes.

## 2. Results and Discussion

### 2.1. Essential Oil Yield

The hydrodistilled leaf essential oils of *Magnolia champaca* accessions were pale yellow in color with a strong odor. The essential oils from 52 accessions of *M. champaca* leaves were extracted, and the mean essential oil yield was determined using one-way ANOVA, followed by Tukey’s HSD test with a 95% confidence interval. A noteworthy difference in EO yield (%) on a fresh weight basis ranging from 0.06% to 0.31% (*v*/*w*) was observed among the populations of *M. champaca* (Table 1). The EO yield of accession MCL27 (0.31% *v*/*w*), collected from the Khordha district, was found to be the highest, followed by accessions MCL9 (0.29% *v*/*w*) and MCL32 (0.26% *v*/*w*) of Ganjam and Khordha district respectively. Similarly, the lowest essential oil yield was found in the accessions MCL3 and MCL4 (0.06% *v*/*w*) which were collected from the Bhadrak district. The essential oil yield of *M. champaca* in the present study was higher than that of the previous report [24], which reported an EO yield of 0.04% in fresh leaves collected from Brazil in different seasons. Further, the resulting variations in the essential oil yield might be due to seasonal variation, geographical location, genetic and environmental factors, etc., as reported in other species [2,3,4]. Our finding showing intraspecific variation in essential oil yield in *M. champaca* is in agreement with reports available in other species such as *Croton gratissimus* [26], *Hypericum gaitii* [3], *Hedychium coronarium* [27] and *Curcuma longa* [10], etc.

### 2.2. Essential Oil Composition

**Figure 1:**
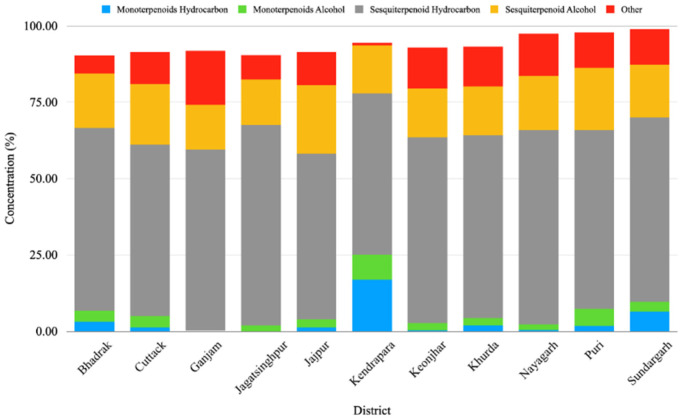
Relative percentages of chemical constituents’ classes in the essential oil of *Magnolia champaca* from different regions of Odisha, India.

The GC-MS analysis of the leaf EO of different *M. champaca* populations collected from eleven districts of Odisha showed wide heterogeneity in chemical constituents. The study revealed a total of 65 compounds through GC-MS analysis, showcasing 90.23 to 98.90% of the total oil composition. The compounds detected through the Elite-5 MS column are listed based on their elution order (Table 2). In all accessions, sesquiterpenes (68.58 to 81.44%) were found to be the major chemical class followed by monoterpenes (0.8 to 25.04%). To be specific, EOs were dominated by mostly sesquiterpene hydrocarbons (52.83 to 65.63%) followed by sesquiterpene alcohol (14.71 to 22.45%) (Table 2, Figure 1). The present result is also consistent with the previous literature, where the leaf essential oil of *M. champaca* collected from Brazil was rich in sesquiterpene hydrocarbons followed by oxygenated sesquiterpenes [24]. In all the populations, the major constituents were β-elemene, γ-muurolene, β-caryophyllene, germacrene B, n-hexadecanol, α-humulene, viridiflorene, bicyclogermacrene, methyl linoleate, linalool, etc. (Table 3). β-elemene (0.23 to 38.76%) (Table 3) was found to be the major constituent of the accessions collected from Jagatsinghpur, Puri, Cuttack, Khordha, Nayagarh, and Ganjam districts (Table 2). γ-muurolene (0.31 to 22.48%) (Table 3) was found to be the predominant constituent of the accessions collected from the Sundargarh and Keonjhar districts (Table 2). Likewise, β-caryophyllene (0.03 to 20.70%) (Table 3) was the major constituent of the accessions of the Jajpur, Bhadrak, and Kendrapara districts (Table 2). The occurrence of three different types of major constituents in different accessions might be due to the difference in geographical location, genetic and environmental factors prevailing at different germplasm collection sites. The phytoconstituents identified herewith have already been reported in the leaf essential oil of different *Magnolia* species [24,25,28,29,30].

Although several phytoconstituents were restricted to one or a small number of populations, the majority were found in varying concentrations in different accessions (Table 2). For example, α-fenchene (0.22–0.79%) was observed only in populations of Kendrapara and Sundargarh districts. Similarly, α-pinene (0.15–0.58%), myrcene (0.17–0.6%), and (E)-β-ocimene (0.27–3.82%) were detected in populations collected from Khordha, Kendrapara and Sundargarh district, however, they were completely absent in the other groups. Our finding showing intraspecific variation in essential oil composition in *M. champaca* is in agreement with reports available in other species such as *Croton gratissimus* [26], *Myristica fragrans* [32], *Hypericum gaitii* [3], *Hedychium coronarium* [27] and *Curcuma longa* [10], etc. The variation in phytoconstituent levels might be due to the place of occurrence of the plant populations, soil characteristics, and predominant environmental factors, of which rainfall and temperature are important parameters [2,3]. As reported [33], the distribution of essential oil chemotypes is related to abiotic factors (temperature, moisture, topography, and edaphic factors) of the region which act on genes of the terpenoid biosynthetic pathways and contribute to the development of diverse essential oil profiles. The chemical diversity observed among *M. champaca* populations resulting from bioclimatic and geographical variables necessitates designing suitable conservation and sustainable utilization strategies, by considering these aspects. Furthermore, all populations representing different chemotypes need to be conserved utilizing different conservation approaches [3,34].

### 2.3. Multivariate Analysis of phytoconstituents

Chemometrics analyses such as PCA, PLS-DA, and sPLS-DA were performed to determine the chemotypes and cluster analyses such as hierarchical clustering, i.e., dendrogram and partitional clustering, i.e., K-means to understand the associations among studied populations of *M. champaca* concerning their EOs’ composition and contents.

**Figure 2:**
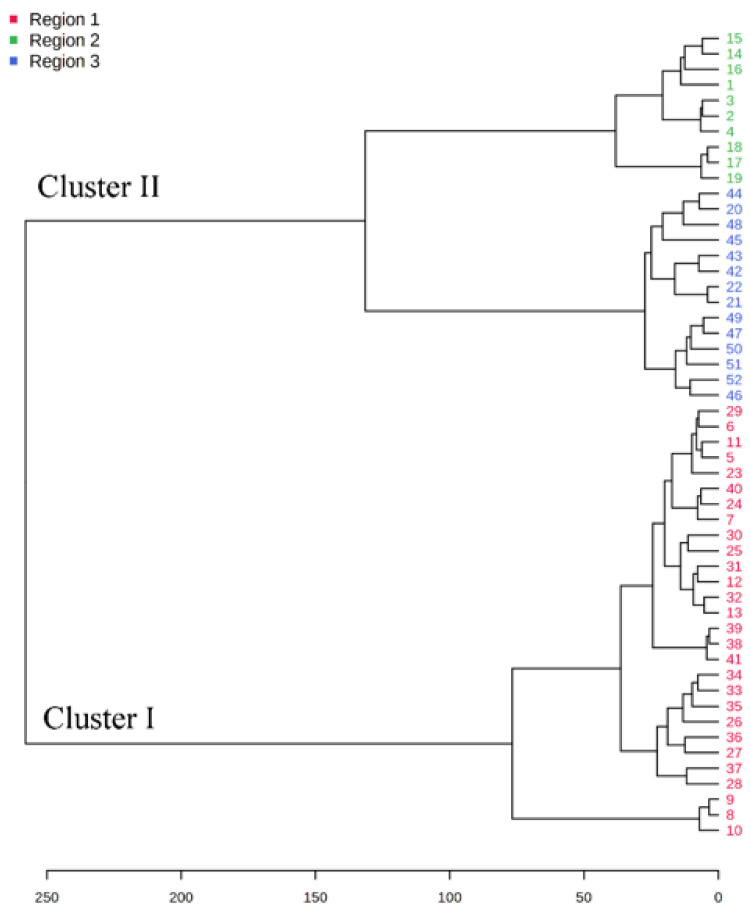
Dendrogram illustrating cluster analysis (distance measure and clustering algorithm were. done using Euclidean and Ward). *M. champaca* accessions are indicated by names according to Table 1.

**Figure 3:**
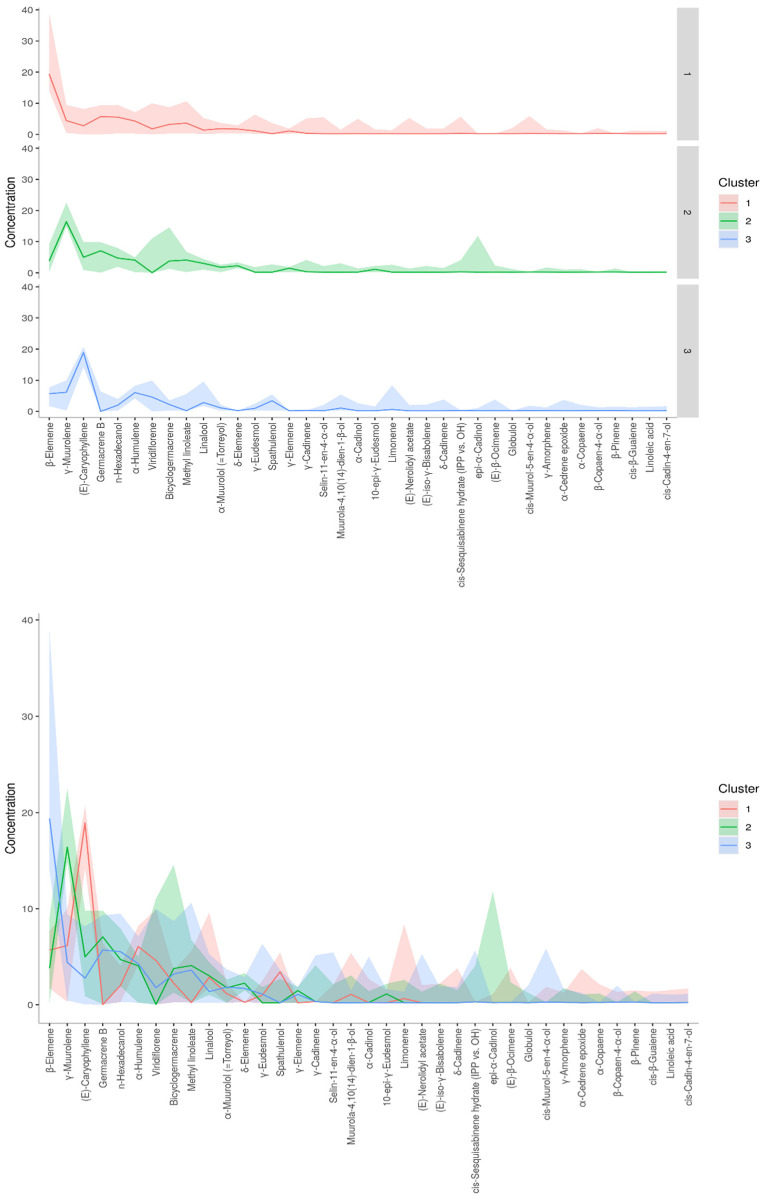
K-means cluster analysis. The X-axis represents phytoconstituents and the Y-axis represents its relative intensities. The median intensities of corresponding clusters are shown in different colored lines.

A dendrogram was constructed based on the Ward linkage-clustering algorithm method using Euclidean distance measures between groups. Concerning the constructed dendrogram, the studied populations were separated into two major clusters, i.e., cluster I and cluster II (Figure 2). Cluster I included the populations collected from Jagatsinghpur, Cuttack, Puri, Khordha, Nayagarh, and Ganjam districts and is characterized by a high content of β-elemene (14.28–38.80%). Cluster II was further subdivided into two subclusters (cluster IIA and IIB) representing two distinct chemotypes. The subcluster IIA included the populations collected from Sundargarh and Keonjhar districts and is characterized by a high content of γ-muurolene (14.92–22.50%) whereas the subcluster IIB included the populations collected from Jajpur, Bhadrak and Kendrapara district and was rich in β-caryophyllene (14.22–20.74%). The variations in the composition of the essential oils in plant species and their principal compounds are possibly because of the changes in abiotic factors such as moisture, temperature and topography which regulate the terpene biosynthesis pathway. This alteration in the biosynthetic pathway can lead to the occurrence of new chemotypes in a plant species [2,35]. Also, the variations in the constituents of the EOs might be connected with microclimatic criteria such as temperature, precipitation or phenological state, which are found to be different in the collection site of *M. champaca* and are possible explanation to alter the oil phytochemical compositions [24,36,37]. Further K-means cluster analysis was prepared by taking the components of EO (variable indices) on the X-axis and their relative intensities on the Y-axis (Figure 3). Different colors represent different clusters and their concentrations. The lines are the median intensities of corresponding clusters. Cluster 1 (red), cluster 2 (green), and cluster 3 (blue) represent the accessions rich in β-elemene, γ-muurolene, and β-caryophyllene, respectively.

**Figure 4:**
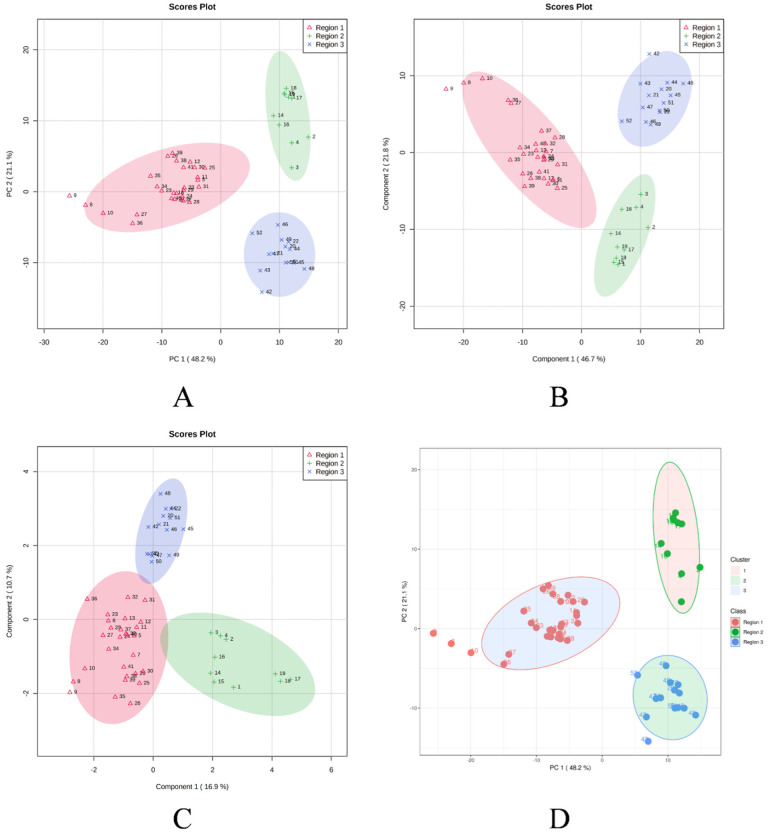
Score plot generated between the selected PCs with their variances. (**A**) PCA; (**B**) PLS-DA; (**C**) sPLS-DA; (**D**) K-means clustering.

**Figure 5:**
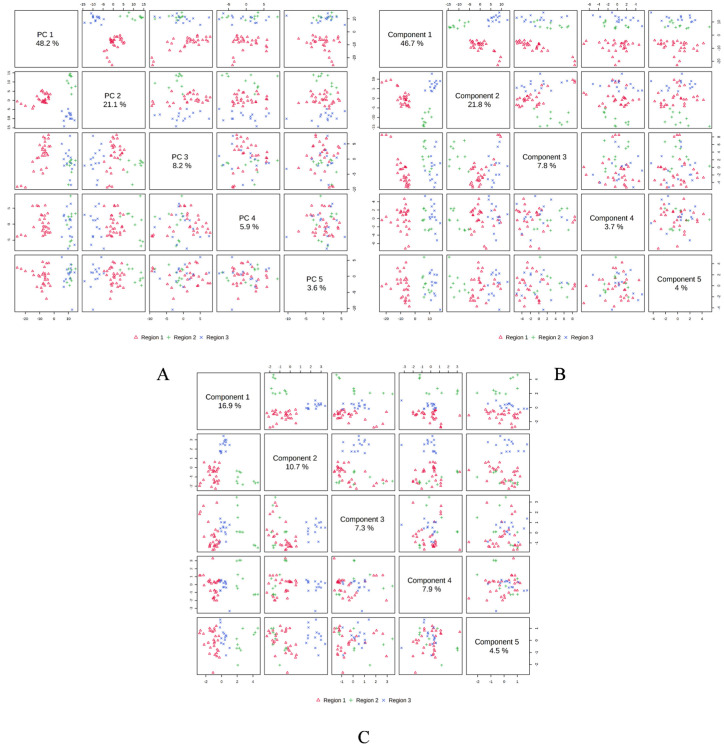
Pairwise score plots between the 5 selected components with their variances shown in the corresponding diagonal cell. (**A**) PCA; (**B**) PLSDA; (**C**) sPLS-DA.

In order to determine the linkages between the *M. champaca* populations, a PCA analysis was performed based on the composition of the essential oils. PCA divided the populations into three groups, confirming the dendrogram’s clustering structure. PCA revealed a total of five principal components (PC) explaining approximately 86.90% of the overall variance. In the score plot, the two principal components (PC1 and PC2) that account for the maximum variation of 69.30% (amongst the populations under study) are shown. (Figure 4). Similarly, score plots generated by PLS-DA, sPLS-DA and K-means clustering show similar distinctions within PCs. Cumulatively pairwise score plots generated through PCA, PLS-DA, and sPLS-DA show a similar trend in the distribution of populations according to their variance (Figure 5).

**Figure 6:**
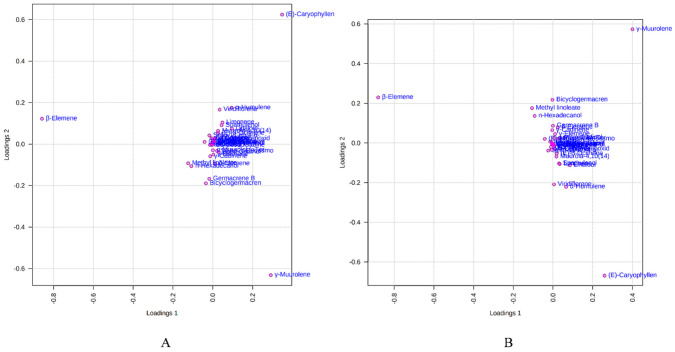
Loadings plot between the selected PCs (PC1 vs. PC2) showing the involvement of major essential oil components (>1%) in the grouping of *M. champaca* accessions. (**A**) PCA; (**B**) PLS-DA.

**Figure 7:**
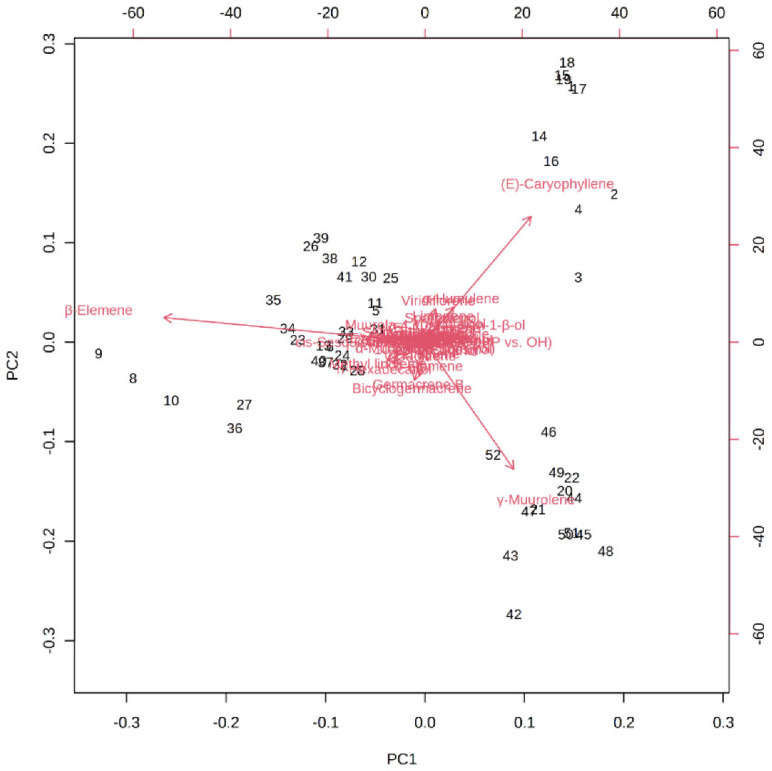
PCA biplot between the selected PCs (PC1 vs. PC2) showing the involvement of major essential oil components (>1%) in the grouping of *M. champaca* accessions. *M. champaca* accessions are indicated by names according to Table 1.

Figure 6 depicts the loading plot of PC1 and PC2 illustrating the contribution of significant EO elements (>1%) in the *M. champaca* populations. The first principal component (PC1) accounts for up to 48.20% variation and had a positive correlation with γ-muurolene and β-caryophyllene (>0.20) but had a negative correlation with β-elemene (>−0.80). Whereas the second principal component (PC2) could explain 21.10% of the total variation and showed a negative correlation with γ-muurolene (>−0.60) and a positive relationship with β-elemene (>0.10) and β-caryophyllene (>0.60). Further biplot (Figure 7) was generated from the loading plots (Figure 6) by following the centering and normalization scaling method. Here the biplot was generated between two principal components, i.e., PC1 and PC2 which once again proved the three chemotypes with major constituents β-elemene, γ-muurolene and β-caryophyllene.

**Figure 8:**
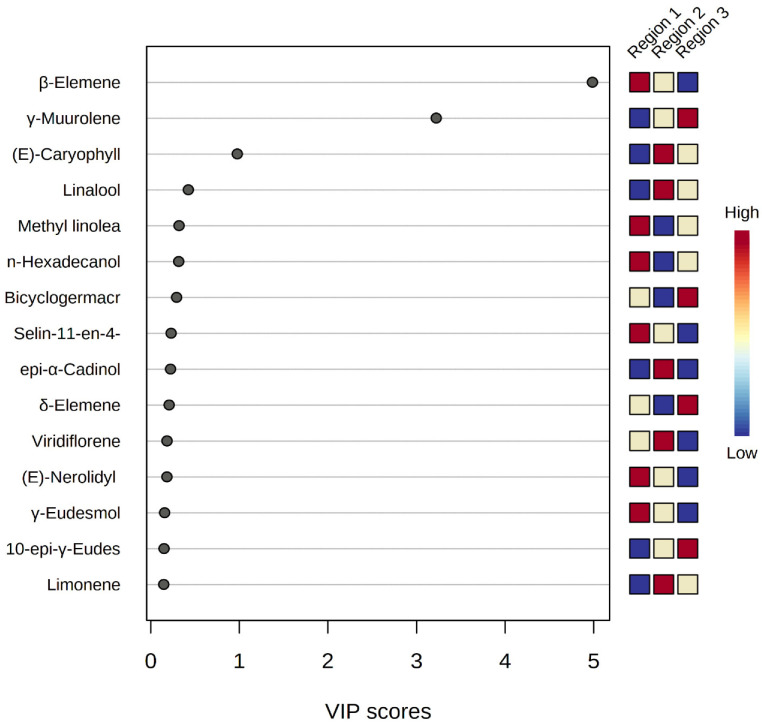
PLS-DA-VIP score plot showing important features of the major constituents of *M. champaca* leaf essential oil. The colored boxes on the right indicate the relative concentrations of the corresponding metabolite in each group under study.

The resulting VIP score plot (Figure 8) represented the strength of each peak distinguishing *M. champaca* samples from different geographical origins. Populations with Variable Importance for the Projection (VIP) > 1 have more influence on population discrimination. The colored boxes against the plot indicate relative concentrations of the corresponding metabolite in each region. The phytoconstituent β-elemene, leading with a VIP score of > 5, is found to be dominant in the accessions of Jagatsinghpur, Cuttack, Puri, Khordha, Nayagarh and Ganjam districts, followed by γ-muurolene, with a score of >3, is found to be highest in the accessions of Sundargarh and Keonjhar district. Furthermore, β-caryophyllene, with a score of >1, is found to be the leading compound of *M. champaca* populations collected from the Jajpur, Bhadrak and Kendrapara districts.

Due to the diverse geographic origin of *M. champaca* accessions, the variances in the phyto-constituents are quite normal. Geographical differences expose the species to a variety of exogenous factor-related stresses that result in the development of a variety of secondary metabolites for their defense [38]. There are reports on other species where the variations in phytoconstituents of different eco-regions were explored by showing the effect of different genetic, climatic and edaphic factors on the variation of essential oil yield and its quality [3,26,27,32]. For the first time, the chemical diversity of leaf essential oil of *M. champaca* has been studied in association with different chemometric approaches such as clustering analysis, PCA, PLS-DA, and sPLS-DA. The chemometric approach has also been effectively used to analyze the correlation and variation in essential oil composition of other species [3,26,27,32].

## 3. Materials and Methods

### 3.1. Plant Material

**Figure 9:**
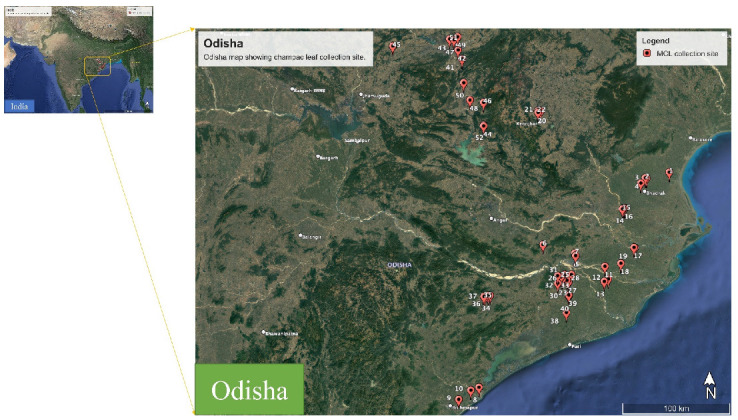
Map showing different regions of sample collection from Odisha, India.

Plant leaves of *M. champaca* were collected from different regions of Odisha in the months of February–August 2022 (Figure 9). The botanical identification of the species was done with the help of the literature and authenticated by Prof. Pratap Chandra Panda, Taxonomist, Centre for Biotechnology, Siksha ‘O’ Anusandhan (Deemed to be University), Bhubaneswar, Odisha, India. The herbarium specimens were prepared and deposited at the Herbarium of the Centre for Biotechnology, as a voucher. The geocoordinates of the areas of occurrence of plant accessions were logged by the GPS and the details are provided in Table 1.

### 3.2. Extraction of Essential Oils

For the isolation of the essential oil, fresh mature leaves measuring 150 g from each accession were cleaned and finely chopped before being subjected to hydrodistillation utilizing a Clevenger-type apparatus for 5 h. EO isolation from each accession was done in three replications to confirm the yield reproducibility. The EO was collected and dried over anhydrous sodium sulfate (Na_2_SO_4_). The EO, was stored in a glass vial at 4 ℃ for further GC-MS analysis. The EO’s yield was calculated following the equation:

% Yield of oil (*v*/*w*) = [volume of oil (in mL)/weight of fresh sample (in g)] × 100

### 3.3. GC-MS Analysis of Isolated EOs

For the analysis of the EOs of *M. champaca*, a gas chromatograph (Clarus 580, PerkinElmer, Waltham, MA, USA) attached to an SQ8S mass spectrometric detector was utilized where 99.99% pure helium was taken as mobile phase (flow rate = 1 mL/min). The EO obtained above was analyzed by injecting 0.1 μL of neat EO into the GC-MS. Separation was carried out on an Elite-5 MS column (30 m in length × 0.25 mm I.D., film thickness 0.25 μm). The oven temperature in the GC-MS was programmed as follows: 60 °C for 0 min, heated at 3 ℃/min to 220 ℃ with 7 min hold, and the total run time was set for 60.33 min. The source and transfer interface temperatures were set at 150 °C and 250 °C respectively. Scanning was performed over a mass scan range of 50–600 *m*/*z* with electron ionization mode at an ionization voltage of 70 eV. Turbo mass TM software 6.1 was utilized to obtain the mass spectra and ion chromatogram.

Identification of each constituent was performed based on the RI (Retention Index) determined by co-injecting a homologous series of straight chain n-alkanes (C_8_–C_20_) run under similar experimental conditions and by comparing calculated RI values with the literature values [31]. The identification of constituents was further confirmed by matching their mass spectra with the inbuilt NIST MS library (NIST 08). Quantification of the essential oil constituents was performed based on relative area percentages.

### 3.4. Statistical Analysis

To compare the statistical differences in EO yield (%) and quality amongst *M. champaca* populations, one-way ANOVA followed by the Tukey HSD test at a 95% confidence level was performed using the statistical software Minitab 17. To investigate the similarity and relationship among *M. champaca* populations based on EO chemical constituents, chemometrics analyses such as Principal Component Analysis (PCA), Partial Least Squares—Discriminant Analysis (PLS-DA), and Sparse Partial Least Squares—Discriminant Analysis (sPLS-DA), and cluster analyses such as hierarchical clustering, i.e., dendrogram and partitional clustering, i.e., K-means clustering was used. The chemometric analysis such as PCA, PLS-DA, sPLS-DA, dendrogram, and K-means clustering was performed using the MetaboAnalyst 5.0, a comprehensive web-based metabolomics analysis tool (https://www.metaboanalyst.ca/ accessed on 21 September 2022). For the chemometrics analysis phytoconstituents with a peak area >1% in at least a single population were chosen as variables. Euclidean distance was used to measure the dissimilarity between populations, and Ward’s variance-minimizing method was performed for hierarchical clustering [39,40].

## 4. Conclusions

This is the first report on the chemical diversity analysis of the leaf essential oil of *Magnolia champaca.* This research revealed significant quantitative and qualitative variations in the chemical profile of the *M. champaca* leaf essential oils from eleven districts of Odisha. Chemometrics techniques could effectively classify the accessions into three different chemotypes rich with β-elemene, β-caryophyllene and γ-muurolene. The result demonstrated that the chemical composition and yield of the essential oil of *M. champaca* might be influenced by the geographical origin of populations and environmental factors. The chemical polymorphism analyzed in the studied populations would facilitate the selection of chemotypes with specific compounds. The chemotypes identified in the *Magnolia champaca* populations could be developed as promising bio-resources for conservation, pharmaceutical application, and further improvement of the taxa.

## Figures and Tables

**Table 1:** Geographical characteristics and essential oil yield of collected *M. champaca* accessions.

Sl No.	Accession No.	Location	District	Latitude	Longitude	Altitude (m)	Oil Yield (%) *	Date of Collection	Voucher Specimen No.
1	MCL1	Basudebpur	Bhadrak	21.132299	86.734740	5	0.11 ± 0.01 ^no^	27 February 2022	2350/CBT
2	MCL2	Charampa	Bhadrak	21.087979	86.520914	16	0.07 ± 0.01 ^pq^	27 February 2022	2351/CBT
3	MCL3	Gelpur	Bhadrak	21.044568	86.480404	16	0.06 ± 0.01 ^q^	28 February 2022	2352/CBT
4	MCL4	Khordanga	Bhadrak	21.084711	86.532225	14	0.06 ± 0.01 ^q^	28 February 2022	2353/CBT
5	MCL5	Chauliaganj	Cuttack	20.456451	85.902186	25	0.20 ± 0.01 ^fghi^	11 March 2022	2354/CBT
6	MCL6	Sankarpur	Cuttack	20.548721	85.618641	42	0.19 ± 0.01 ^ghij^	11 March 2022	2355/CBT
7	MCL7	Zobra	Cuttack	20.478640	85.896844	26	0.22 ± 0.01 ^defg^	11 March 2022	2356/CBT
8	MCL8	Barlapalli	Ganjam	19.385068	85.060824	5	0.25 ± 0.01 ^cd^	23 March 2022	2357/CBT
9	MCL9	Korapalli	Ganjam	19.285533	84.884574	22	0.29 ± 0.01 ^ab^	23 March 2022	2358/CBT
10	MCL10	Chatrapur	Ganjam	19.361912	84.989735	23	0.23 ± 0.01 ^cdef^	23 March 2022	2359/CBT
11	MCL11	Raghunathpur	Jagatsinghpur	20.368431	86.158429	14	0.18 ± 0.01 ^hijk^	8 April 2022	2360/CBT
12	MCL12	Patenigan	Jagatsinghpur	20.264791	86.187224	13	0.17 ± 0.01 ^ijkl^	8 April 2022	2361/CBT
13	MCL13	Benupur	Jagatsinghpur	20.245119	86.152115	12	0.18 ± 0.01 ^hijk^	8 April 2022	2362/CBT
14	MCL14	Ameswarpur	Jajpur	20.841792	86.328084	14	0.11 ± 0.01 ^no^	18 April 2022	2363/CBT
15	MCL15	Baidyarajpur	Jajpur	20.834910	86.315929	16	0.12 ± 0.01 ^mno^	18 April 2022	2364/CBT
16	MCL16	Kalyanpur	Jajpur	20.840515	86.336724	17	0.12 ± 0.01 ^mno^	18 April 2022	2365/CBT
17	MCL17	Gulnagar	Kendrapara	20.519592	86.414565	5	0.14 ± 0.01 ^lmn^	4 May 2022	2366/CBT
18	MCL18	Tarada	Kendrapara	20.390703	86.298042	11	0.16 ± 0.01 ^jkl^	4 May 2022	2367/CBT
19	MCL19	Garapur	Kendrapara	20.516980	86.423092	6	0.17 ± 0.01 ^ijkl^	4 May 2022	2368/CBT
20	MCL20	Raintira	Keonjhar	21.624656	85.579860	486	0.23 ± 0.01 ^cdef^	25 May 2022	2369/CBT
21	MCL21	Dhenkpur	Keonjhar	21.636924	85.603657	476	0.19 ± 0.01 ^ghij^	25 May 2022	2370/CBT
22	MCL22	Bidyarajsahi	Keonjhar	21.635674	85.575923	496	0.20 ± 0.01 ^fghi^	25 May 2022	2371/CBT
23	MCL23	Forest park	Khordha	20.257316	85.826255	36	0.10 ± 0.01 ^op^	3 June 2022	2372/CBT
24	MCL24	Khandagiri	Khordha	20.264246	85.783965	63	0.18 ± 0.01 ^hijk^	3 June 2022	2373/CBT
25	MCL25	Durgamadhab nagar	Khordha	20.291257	85.775147	72	0.21 ± 0.01 ^efgh^	3 June 2022	2374/CBT
26	MCL26	Jaydev vihar	Khordha	20.302974	85.805600	62	0.09 ± 0.01 ^opq^	11 June 2022	2375/CBT
27	MCL27	Old town	Khordha	20.241975	85.839274	20	0.31 ± 0.02 ^a^	11 June 2022	2376/CBT
28	MCL28	Palasuni	Khordha	20.303605	85.865650	21	0.12 ± 0.01 ^mno^	11 June 2022	2377/CBT
29	MCL29	Nayapalli	Khordha	20.303781	85.805565	65	0.17 ± 0.01 ^ijkl^	15 June 2022	2378/CBT
30	MCL30	Tomando	Khordha	20.233206	85.745459	29	0.22 ± 0.01 ^defg^	15 June 2022	2379/CBT
31	MCL31	Gothapatna	Khordha	20.294390	85.743991	78	0.21 ± 0.01 ^efgh^	15 June 2022	2380/CBT
32	MCL32	Sampur	Khordha	20.284518	85.773200	72	0.26 ± 0.01 ^bc^	24 June 2022	2381/CBT
33	MCL33	Kalinga nagar	Khordha	20.284124	85.775564	75	0.24 ± 0.01 ^cde^	24 June 2022	2382/CBT
34	MCL34	Itamati nagar	Nayagarh	20.136615	85.156632	71	0.19 ± 0.01 ^ghij^	13 July 2022	2383/CBT
35	MCL35	Khandapada	Nayagarh	20.128100	85.104011	106	0.15 ± 0.01 ^klm^	13 July 2022	2384/CBT
36	MCL36	Pratapprasad	Nayagarh	20.136224	85.140644	76	0.20 ± 0.01 ^fghi^	16 July 2022	2385/CBT
37	MCL37	Old town	Nayagarh	20.117820	85.110669	89	0.16 ± 0.01 ^jkl^	16 July 2022	2386/CBT
38	MCL38	Siriapur	Puri	19.993425	85.820178	9	0.20 ± 0.01 ^fghi^	21 July 2022	2387/CBT
39	MCL39	Pipili	Puri	20.135786	85.841370	14	0.21 ± 0.01 ^efgh^	21 July 2022	2388/CBT
40	MCL40	Samalapur	Puri	20.127881	85.840332	13	0.19 ± 0.01 ^ghij^	21 July 2022	2389/CBT
41	MCL41	Chandanpur	Puri	19.886747	85.814705	17	0.12 ± 0.01 ^mno^	22 July 2022	2390/CBT
42	MCL42	Lathikata	Sundargarh	22.136464	84.878344	186	0.09 ± 0.01 ^opq^	27 July 2022	2391/CBT
43	MCL43	Udit nagar	Sundargarh	22.221226	84.841473	196	0.11 ± 0.01 ^no^	27 July 2022	2392/CBT
44	MCL44	Tensa	Sundargarh	21.515307	85.102925	257	0.09 ± 0.01 ^opq^	27 July 2022	2393/CBT
45	MCL45	Badagaon	Sundargarh	22.169503	84.301875	252	0.11 ± 0.01 ^no^	28 July 2022	2394/CBT
46	MCL46	Fuljhar	Sundargarh	21.707314	85.102838	219	0.23 ± 0.01 ^cdef^	28 July 2022	2395/CBT
47	MCL47	Basanti	Sundargarh	22.221585	84.841456	196	0.11 ± 0.01 ^no^	28 July 2022	2396/CBT
48	MCL48	Ruguda	Sundargarh	21.726694	84.982909	155	0.18 ± 0.01 ^hijk^	3 August 2022	2397/CBT
49	MCL49	Sector 4	Sundargarh	22.245085	84.874139	211	0.23 ± 0.01 ^cdef^	3 August 2022	2398/CBT
50	MCL50	Rajamunda	Sundargarh	21.867753	84.928222	163	0.12 ± 0.01 ^mno^	4 August 2022	2399/CBT
51	MCL51	Panposh	Sundargarh	22.226728	84.804252	188	0.09 ± 0.01 ^opq^	4 August 2022	2400/CBT
52	MCL52	Tensa	Sundargarh	21.522735	85.101525	444	0.18 ± 0.01 ^hijk^	4 August 2022	2401/CBT

The essential oil yield is given in % (*v*/*w*) based on the fresh weight of the plant material, i.e., in mL/100 g fresh weight. The mean having different letters in a column was significantly different according to Tukey’s HSD test at *p* < 0.05. * Each sample was analyzed in triplicate.

**Table 2:** Relative contents of each constituent in *Magnolia champaca* leaf essential oil from Bhadrak (N = 4), Cuttack (N = 3), Ganjam (N = 3), Jajpur (N = 3), Jagatsinghpur (N = 3), Kendrapara (N = 3), Keonjhar (N = 3), Khordha (N = 11), Nayagarh (N = 4), Puri (N = 4), Sundargarh (N = 11).

Sl No.	Compounds	RI ^a^	RI ^b^	Peak Area (%) *										
				Bhadrak	Cuttack	Ganjam	Jagatsinghpur	Jajpur	Kendrapara	Keonjhar	Khordha	Nayagarh	Puri	Sundargarh
1	(3E)-Hexenol	848	853	0.10 ± 0.01 ^c^	0.17 ± 0.05 ^a^	-	-	-	0.16 ± 0.07 ^ab^	-	0.14 ± 0.05 ^b^	0.10 ± 0.01 ^c^	-	0.14 ± 0.06 ^b^
2	α-Pinene	928	939	-	-	-	-	-	0.58 ± 0.06 ^a^	-	0.15 ± 0.03 ^c^	-	-	0.35 ± 0.17 ^b^
3	α-Fenchene	945	952	-	-	-	-	-	0.79 ± 0.06 ^a^	-	-	-	-	0.22 ± 0.09 ^b^
4	Sabinene	967	975	-	0.11 ± 0.06 ^c^	-	-	-	1.90 ± 0.09 ^a^	-	0.18 ± 0.03 ^c^	-	-	0.32 ± 0.20 ^b^
5	β-Pinene	973	979	-	0.17 ± 0.08 ^d^	-	-	-	1.49 ± 0.09 ^b^	-	0.29 ± 0.11 ^c^	-	-	1.68 ± 0.43 ^a^
6	Myrcene	982	990	-	-	-	-	-	0.60 ± 0.06 ^a^	-	0.17 ± 0.04 ^b^	-	-	0.19 ± 0.03 ^b^
7	Limonene	1028	1029	-	0.45 ± 0.28 ^c^	-	-	1.25 ± 0.03 ^b^	7.36 ± 1.00 ^a^	-	0.11 ± 0.05 ^d^	-	1.14 ± 0.06 ^b^	1.11 ± 0.85 ^b^
8	(E)-β-Ocimene	1040	1050	-	-	-	-	-	3.82 ± 0.09 ^a^	-	0.27 ± 0.15 ^c^	-	-	1.86 ± 0.72 ^b^
9	Linalool	1096	1096	3.67 ± 0.18 ^c^	3.53 ± 0.18 ^c^	0.80 ± 0.07 ^g^	1.87 ± 0.09 ^f^	2.33 ± 0.12 ^ef^	7.59 ± 0.38 ^a^	2.40 ± 0.12 ^e^	2.26 ± 0.11 ^ef^	1.88 ± 0.09 ^ef^	5.24 ± 0.26 ^b^	2.95 ± 0.15 ^d^
10	γ-Terpineol	1176	1199	-	0.17 ± 0.03 ^d^	-	-	0.23 ± 0.01 ^c^	0.61 ± 0.06 ^a^	-	0.17 ± 0.08 ^d^	-	0.35 ± 0.02 ^b^	0.19 ± 0.09 ^d^
11	Linalool formate	1191	1216	-	0.21 ± 0.08 ^a^	-	-	-	0.20 ± 0.03 ^a^	-	0.13 ± 0.05 ^c^	-	0.16 ± 0.01 ^b^	0.16 ± 0.03 ^b^
12	Menthyl acetate	1291	1295	1.46 ± 0.04 ^a^	0.13 ± 0.01 ^c^	-	-	-	-	0.13 ± 0.01 ^c^	0.30 ± 0.16 ^b^	0.16 ± 0.03 ^c^	0.25 ± 0.02 ^b^	0.24 ± 0.05 ^b^
13	iso-Menthyl acetate	1306	1305	1.56 ± 0.02 ^a^	0.14 ± 0.01 ^de^	-	-	-	0.11 ± 0.07 ^e^	0.15 ± 0.02 ^de^	0.31 ± 0.18 ^b^	0.20 ± 0.04 ^cd^	0.26 ± 0.02 ^bc^	0.30 ± 0.07 ^b^
14	δ-Elemene	1328	1338	1.99 ± 0.01 ^bcd^	2.22 ± 0.56 ^b^	-	1.75 ± 0.04 ^d^	1.95 ± 0.05 ^cd^	0.24 ± 0.08 ^f^	2.63 ± 0.58 ^a^	1.89 ± 0.52 ^cd^	1.74 ± 0.64 ^d^	2.02 ± 0.08 ^bc^	1.36 ± 0.92 ^e^
15	α-Copaene	1367	1376	0.12 ± 0.05 ^d^	0.28 ± 0.25 ^c^	1.49 ± 0.07 ^b^	0.23 ± 0.05 ^cd^	1.62 ± 0.05 ^b^	2.11 ± 0.09 ^a^	0.28 ± 0.05 ^c^	0.28 ± 0.12 ^c^	0.22 ± 0.09 ^cd^	0.34 ± 0.02 ^c^	1.59 ± 0.33 ^b^
16	β-Elemene	1383	1390	3.69 ± 0.18 ^f^	15.93 ± 0.80 ^d^	35.76 ± 1.79 ^a^	18.51 ± 0.93 ^c^	5.72 ± 0.29 ^ef^	6.67 ± 0.33 ^e^	3.42 ± 0.17 ^f^	18.30 ± 0.92 ^cd^	22.61 ± 1.13 ^b^	19.11 ± 0.96 ^c^	3.67 ± 0.18 ^c^
17	(Z)-Caryophyllene	1399	1408	-	1.79 ± 0.94 ^a^	0.12 ± 0.04 ^d^	0.23 ± 0.02 ^c^	-	-	0.12 ± 0.01 ^d^	0.22 ± 0.21 ^c^	0.27 ± 0.18 ^c^	-	0.38 ± 0.21 ^b^
18	β-Caryophyllene	1412	1419	18.20 ± 0.91 ^a^	1.00 ± 0.05 ^f^	1.64 ± 0.08 ^ef^	5.46 ± 0.27 ^c^	18.79 ± 0.94 ^a^	18.73 ± 0.94 ^a^	8.57 ± 0.43 ^b^	3.55 ± 0.18 ^d^	2.93 ± 0.15 ^de^	5.49 ± 0.27 ^c^	4.00 ± 0.20 ^cd^
19	γ-Elemene	1421	1436	1.76 ± 0.02 ^b^	1.87 ± 0.65 ^ab^	0.30 ± 0.05 ^d^	1.17 ± 0.32 ^c^	1.05 ± 0.05 ^c^	0.14 ± 0.07 ^d^	1.70 ± 0.09 ^b^	1.19 ± 0.33 ^c^	1.02 ± 0.20 ^c^	1.16 ± 0.06 ^c^	1.97 ± 0.68 ^a^
20	Aromadendrene	1427	1441	0.13 ± 0.04 ^d^	0.18 ± 0.08 ^b^	0.07 ± 0.01 ^e^	0.12 ± 0.01 ^d^	-	0.12 ± 0.07 ^d^	-	0.15 ± 0.06 ^c^	-	-	0.21 ± 0.12 ^a^
21	6,9-Guaiadiene	1434	1444	0.13 ± 0.04 ^f^	0.27 ± 0.01 ^e^	-	0.35 ± 0.06 ^de^	1.38 ± 0.03 ^b^	-	1.65 ± 0.07 ^a^	0.34 ± 0.11 ^de^	0.33 ± 0.11 ^de^	0.48 ± 0.04 ^c^	0.40 ± 0.13 ^cd^
22	cis-Muurola-3,5-diene	1439	1450	0.14 ± 0.04 ^c^	0.22 ± 0.02 ^b^	0.12 ± 0.04 ^c^	0.28 ± 0.03 ^b^	0.23 ± 0.01 ^b^	-	1.36 ± 0.05 ^a^	0.25 ± 0.07 ^b^	0.21 ± 0.05 ^b^	0.23 ± 0.01 ^b^	0.26 ± 0.11 ^b^
23	α-Humulene	1446	1454	6.42 ± 0.32 ^b^	4.24 ± 0.21 ^de^	-	6.07 ± 0.30 ^bc^	7.36 ± 0.37 ^a^	5.40 ± 0.27 ^c^	3.86 ± 0.19 ^e^	4.68 ± 0.23 ^d^	4.06 ± 0.20 ^de^	2.92 ± 0.15 ^f^	4.27 ± 0.21 ^de^
24	allo-Aromadendrene	1452	1460	1.14 ± 0.04 ^a^	0.17 ± 0.05 ^de^	-	0.19 ± 0.06 ^cd^	0.20 ± 0.01 ^cd^	0.13 ± 0.07 ^e^	0.20 ± 0.02 ^cd^	0.22 ± 0.07 ^bcd^	0.22 ± 0.05 ^bcd^	0.24 ± 0.01 ^bc^	0.26 ± 0.15 ^b^
25	cis-Cadina-1(6),4-diene	1464	1463	1.52 ± 0.02 ^a^	-	0.36 ± 0.05 ^b^	-	-	0.18 ± 0.03 ^c^	-	0.21 ± 0.08 ^c^	-	-	0.23 ± 0.02 ^c^
26	γ-Gurjunene	1466	1477	-	1.82 ± 0.04 ^a^	0.15 ± 0.04 ^de^	1.69 ± 0.09 ^a^	1.70 ± 0.05 ^a^	0.20 ± 0.03 ^d^	-	0.38 ± 0.13 ^c^	1.51 ± 0.26 ^b^	0.27 ± 0.02 ^cd^	0.25 ± 0.11 ^cd^
27	γ-Muurolene	1481	1479	7.04 ± 0.35 ^b^	3.48 ± 0.17 ^e^	8.29 ± 0.41 ^b^	5.37 ± 0.27 ^cd^	4.29 ± 0.21 ^de^	5.66 ± 0.28 ^c^	16.62 ± 0.83 ^a^	4.85 ± 0.24 ^cd^	4.79 ± 0.24 ^cd^	3.34 ± 0.17 ^e^	17.50 ± 0.87 ^a^
28	β-Selinene	1479	1490	1.67 ± 0.02 ^a^	1.68 ± 0.25 ^a^	1.08 ± 0.09 ^b^	1.74 ± 0.04 ^a^	-	0.33 ± 0.08 ^c^	-	1.58 ± 0.19 ^a^	-	-	1.58 ± 0.13 ^a^
29	cis-β-Guaiene	1484	1493	0.19 ± 0.06 ^d^	1.50 ± 0.09 ^b^	-	0.24 ± 0.06 ^d^	1.15 ± 0.08 ^c^	0.22 ± 0.03 ^d^	1.48 ± 0.07 ^b^	0.26 ± 0.17 ^d^	1.76 ± 0.16 ^a^	1.48 ± 0.04 ^b^	1.56 ± 0.37 ^b^
30	Viridiflorene	1487	1496	2.59 ± 0.13 ^e^	7.95 ± 0.40 ^b^	1.56 ± 0.08 ^f^	6.37 ± 0.32 ^c^	4.53 ± 0.23 ^d^	8.81 ± 0.44 ^a^	-	3.82 ± 0.19 ^d^	1.09 ± 0.05 ^f^	8.37 ± 0.42 ^ab^	7.00 ± 0.35 ^c^
31	γ-Amorphene	1494	1495	0.31 ± 0.06 ^cd^	0.46 ± 0.05 ^c^	0.17 ± 0.04 ^d^	1.62 ± 0.08 ^a^	-	1.27 ± 0.09 ^b^	-	1.61 ± 0.35 ^a^	1.61 ± 0.08 ^a^	-	1.52 ± 0.56 ^a^
32	Bicyclogermacrene	1498	1500	2.93 ± 0.15 ^ef^	2.66 ± 0.1 ^f^	6.30 ± 0.32 ^b^	3.33 ± 0.17 ^de^	2.37 ± 0.1 ^f^	-	7.36 ± 0.37 ^a^	3.43 ± 0.17 ^de^	5.93 ± 0.30 ^b^	3.59 ± 0.18 ^d^	4.49 ± 0.22 ^c^
33	γ-Cadinene	1504	1513	0.15 ± 0.04 ^e^	0.23 ± 0.09 ^e^	0.21 ± 0.05 ^e^	1.29 ± 0.09 ^d^	-	-	4.11 ± 0.09 ^a^	1.63 ± 1.24 ^c^	4.15 ± 1.69 ^a^	2.60 ± 0.08 ^b^	0.19 ± 0.06 ^e^
34	δ-Cadinene	1509	1523	1.83 ± 0.02 ^b^	1.12 ± 0.65 ^d^	1.47 ± 0.09 ^c^	1.24 ± 0.04 ^d^	0.27 ± 0.03 ^f^	2.46 ± 1.53 ^a^	-	1.98 ± 0.35 ^b^	0.22 ± 0.16 ^f^	0.16 ± 0.01 ^fg^	0.61 ± 0.42 ^e^
35	(E)-iso-γ-Bisabolene	1513	1529	0.15 ± 0.04 ^f^	0.12 ± 0.09 ^f^	0.13 ± 0.04 ^f^	0.17 ± 0.07 ^f^	1.69 ± 0.08 ^b^	0.17 ± 0.07 ^f^	1.01 ± 0.07 ^e^	1.43 ± 0.37 ^c^	1.90 ± 0.31 ^a^	1.26 ± 0.06 ^d^	1.12 ± 0.82 ^de^
36	trans-Cadina-1,4-diene	1521	1534	-	0.16 ± 0.06 ^a^	-	0.17 ± 0.08 ^a^	-	-	0.13 ± 0.01 ^b^	0.12 ± 0.04 ^b^	0.08 ± 0.02 ^c^	-	0.13 ± 0.03 ^b^
37	α-Cadinene	1525	1538	0.28 ± 0.06 ^a^	0.17 ± 0.08 ^b^	0.17 ± 0.05 ^b^	0.16 ± 0.05 ^b^	-	-	0.12 ± 0.01 ^c^	0.12 ± 0.05 ^c^	0.11 ± 0.03 ^c^	-	0.12 ± 0.02 ^c^
38	Hedycaryol	1540	1548	1.51 ± 0.02 ^a^	0.23 ± 0.13 ^de^	-	0.35 ± 0.02 ^c^	1.51 ± 0.03 ^a^	0.62 ± 0.06 ^b^	-	0.24 ± 0.10 ^de^	0.16 ± 0.08 ^e^	-	0.32 ± 0.20 ^cd^
39	Germacrene B	1555	1561	5.54 ± 0.28 ^d^	6.69 ± 0.33 ^bc^	-	7.73 ± 0.39 ^a^	-		6.13 ± 0.31 ^cd^	7.01 ± 0.35 ^ab^	6.71 ± 0.34 ^bc^	5.35 ± 0.27 ^d^	5.45 ± 0.27 ^d^
40	cis-Sesquisabinene hydrate (IPP vs. OH)	1552	1544	1.49 ± 0.02 ^b^	3.16 ± 1.05 ^a^	-	1.51 ± 0.15 ^b^	-	0.37 ± 0.08 ^c^	-	1.67 ± 2.23 ^b^	-	-	0.54 ± 0.05 ^c^
41	cis-Muurol-5-en-4-α-ol	1558	1561	0.20 ± 0.06 ^c^	0.23 ± 0.17 ^c^	-	0.15 ± 0.04 ^c^	0.21 ± 0.01 ^c^	0.13 ± 0.07 ^c^	-	0.15 ± 0.04 ^c^	3.01 ± 4.01 ^a^	-	0.71 ± 0.69 ^b^
42	trans-Dauca-4(11),7-diene	1562	1557	-	-	-	-	-	-	-	0.19 ± 0.05 ^b^	0.12 ± 0.01 ^c^	0.33 ± 0.02 ^a^	-
43	β-Copaen-4-α-ol	1566	1590	1.34 ± 0.03 ^c^	0.39 ± 0.08 ^e^	0.08 ± 0.01 ^g^	0.28 ± 0.06 ^ef^	1.48 ± 0.03 ^b^	0.65 ± 0.06 ^d^	0.20 ± 0.02 ^fg^	0.35 ± 0.18 ^e^	0.36 ± 0.13 ^e^	1.85 ± 0.06 ^a^	0.62 ± 0.61 ^d^
44	α-Cedrene epoxide	1574	1575	-	0.41 ± 0.03 ^d^	0.15 ± 0.04 ^ef^	-	1.40 ± 0.03 ^c^	-	0.27 ± 0.05 ^de^	1.67 ± 0.35 ^b^	1.54 ± 0.04 ^bc^	-	2.61 ± 1.26 ^a^
45	Spathulenol	1571	1578	3.93 ± 0.68 ^a^	1.90 ± 0.96 ^b^	0.09 ± 0.01 ^f^	1.49 ± 0.05 ^cd^	1.55 ± 0.05 ^c^	4.10 ± 1.15 ^a^	1.28 ± 0.05 ^cde^	1.05 ± 0.70 ^e^	0.38 ± 0.18 ^f^	1.95 ± 0.06 ^b^	1.18 ± 0.98 ^de^
46	Globulol	1585	1590	0.24 ± 0.06 ^f^	1.70 ± 0.16 ^a^	-	1.42 ± 0.06 ^c^	1.61 ± 0.08 ^ab^	0.26 ± 0.08 ^f^	1.53 ± 0.07 ^bc^	0.32 ± 0.19 ^f^	0.39 ± 0.18 ^f^	1.23 ± 0.06 ^d^	0.63 ± 0.68 ^e^
47	Guaiol	1595	1600	0.12 ± 0.04 ^e^	0.33 ± 0.13 ^c^	0.09 ± 0.01 ^e^	0.26 ± 0.04 ^cd^	0.25 ± 0.01 ^d^	0.64 ± 0.06 ^b^	0.12 ± 0.01 ^e^	0.15 ± 0.05 ^e^	0.16 ± 0.11 ^e^	1.54 ± 0.04 ^a^	0.31 ± 0.24 ^cd^
48	9,11-epoxy-Guaia-3,10(14)-diene	1599	1601	1.90 ± 0.02 ^a^	-	-	0.16 ± 0.05 ^d^	-	-	-	0.28 ± 0.21 ^c^	0.18 ± 0.04 ^d^	-	0.37 ± 0.19 ^b^
49	10-epi-γ-Eudesmol	1610	1623	1.55 ± 0.03 ^b^	1.90 ± 0.23 ^a^	0.22 ± 0.05 ^e^	1.23 ± 0.05 ^c^	1.83 ± 0.05 ^a^	0.18 ± 0.03 ^e^	1.08 ± 0.09 ^cd^	1.20 ± 0.55 ^c^	1.83 ± 0.34 ^a^	1.76 ± 0.04 ^a^	0.89 ± 0.50 ^d^
50	Muurola-4,10(14)-dien-1-β-ol	1618	1631	1.08 ± 0.03 ^c^	1.83 ± 0.22 ^b^	0.30 ± 0.05 ^e^	1.12 ± 0.02 ^c^	1.05 ± 0.05 ^c^	4.71 ± 0.58 ^a^	1.63 ± 0.07 ^b^	1.02 ± 0.70 ^cd^	1.57 ± 0.03 ^b^	1.01 ± 0.06 ^cd^	0.75 ± 0.36 ^d^
51	γ-Eudesmol	1633	1632	1.79 ± 0.03 ^b^	1.35 ± 0.58 ^e^	1.55 ± 0.07 ^cde^	1.11 ± 0.01 ^f^	1.77 ± 0.08 ^bc^	0.84 ± 0.06 ^g^	1.48 ± 0.07 ^de^	1.03 ± 0.36 ^fg^	1.68 ± 0.28 ^bcd^	1.82 ± 0.08 ^ab^	2.01 ± 1.68 ^a^
52	cis-Cadin-4-en-7-ol	1636	1636	0.18 ± 0.06 ^f^	0.37 ± 0.12 ^e^	1.75 ± 0.07 ^ab^	1.43 ± 0.07 ^c^	1.58 ± 0.05 ^bc^	0.32 ± 0.08 ^ef^	1.76 ± 0.07 ^a^	1.43 ± 0.24 ^c^	0.40 ± 0.21 ^e^	1.57 ± 0.04 ^c^	0.70 ± 0.45 ^d^
53	epi-α-Cadinol	1640	1640	1.09 ± 0.03 ^c^	1.58 ± 0.46 ^b^	-	-	1.50 ± 0.03 ^b^	-	0.18 ± 0.02 ^f^	0.32 ± 0.09 ^e^	0.18 ± 0.07 ^f^	0.49 ± 0.04 ^d^	1.78 ± 4.05 ^a^
54	α-Muurolol (=Torreyol)	1645	1646	1.14 ± 0.03 ^c^	2.85 ± 0.71 ^a^	1.83 ± 0.09 ^b^	2.85 ± 0.58 ^a^	-	1.09 ± 0.06 ^c^	1.72 ± 0.09 ^b^	1.80 ± 0.59 ^b^	1.72 ± 1.81 ^b^	1.97 ± 0.08 ^b^	1.96 ± 0.80 ^b^
55	α-Cadinol	1652	1654	-	-	1.64 ± 0.07 ^cd^	-	2.31 ± 0.08 ^b^	-	1.31 ± 0.09 ^e^	1.86 ± 1.05 ^c^	2.83 ± 0.46 ^a^	1.39 ± 0.08 ^e^	1.52 ± 1.49 ^de^
56	Selin-11-en-4-α-ol	1653	1659	1.84 ± 0.02 ^bc^	1.58 ± 0.41 ^cde^	1.53 ± 0.07 ^de^	1.58 ± 0.03 ^cde^	4.25 ± 1.06 ^a^	1.61 ± 0.09 ^bcd^	1.30 ± 0.05 ^ef^	1.88 ± 0.98 ^b^	1.32 ± 1.78 ^ef^	1.65 ± 0.04 ^bcd^	1.06 ± 0.86 ^f^
57	(Z)-Nerolidyl acetate	1668	1677	-	-	0.11 ± 0.01 ^c^	-	-	0.19 ± 0.03 ^b^	-	0.09 ± 0.01 ^c^	0.09 ± 0.01 ^c^	-	0.66 ± 1.04 ^a^
58	Eudesm-7(11)-en-4-ol	1699	1700	0.15 ± 0.04 ^c^	0.17 ± 0.08 ^c^	1.70 ± 0.07 ^a^	0.18 ± 0.07 ^c^	0.18 ± 0.01 ^c^	0.41 ± 0.08 ^b^	-	0.18 ± 0.06 ^c^	0.18 ± 0.09 ^c^	0.14 ± 0.01 ^c^	0.35 ± 0.18 ^b^
59	(E)-Nerolidyl acetate	1709	1717	1.55 ± 0.02 ^c^	0.21 ± 0.09 ^e^	3.36 ± 0.09 ^a^	0.13 ± 0.03 ^e^	1.45 ± 0.08 ^cd^	0.26 ± 0.08 ^e^	1.24 ± 0.05 ^d^	1.38 ± 0.22 ^cd^	1.40 ± 0.24 ^cd^	1.80 ± 0.06 ^b^	1.29 ± 1.35 ^d^
60	(Z)-Nuciferol	1727	1725	-	-	1.45 ± 0.05 ^a^	-	0.26 ± 0.03 ^bc^	-	-	0.13 ± 0.06 ^d^	-	0.23 ± 0.01 ^c^	0.34 ± 0.23 ^b^
61	γ-Costol	1744	1746	0.28 ± 0.06 ^d^	0.24 ± 0.08 ^de^	0.48 ± 0.05 ^c^	0.23 ± 0.05 ^de^	1.45 ± 0.03 ^a^	-	1.21 ± 0.05 ^b^	0.18 ± 0.07 ^e^	0.31 ± 0.03 ^d^	0.31 ± 0.02 ^d^	0.26 ± 0.09 ^de^
62	n-Hexadecanol	1855	1875	2.51 ± 0.13 ^e^	6.12 ± 0.31 ^b^	6.02 ± 0.30 ^b^	5.56 ± 0.28 ^bc^	2.56 ± 0.13 ^e^	-	7.25 ± 0.36 ^a^	5.15 ± 0.26 ^c^	6.87 ± 0.34 ^a^	5.50 ± 0.28 ^bc^	4.18 ± 0.21 ^d^
63	Manool oxide	1970	1987	-	-	0.09 ± 0.01 ^d^	-	-	-	-	0.12 ± 0.01 ^c^	0.34 ± 0.04 ^a^	-	0.19 ± 0.03 ^b^
64	Methyl linoleate	2055	2085	1.38 ± 0.07 ^f^	3.16 ± 0.16 ^e^	8.08 ± 0.40 ^a^	1.89 ± 0.09 ^f^	3.63 ± 0.18 ^cde^	-	4.62 ± 0.23 ^b^	3.99 ± 0.20 ^c^	4.15 ± 0.21 ^bc^	3.76 ± 0.19 ^cd^	3.27 ± 0.16 ^de^
65	Linoleic acid	2111	2133	0.27 ± 0.06 ^ef^	0.40 ± 0.25 ^de^	1.88 ± 0.09 ^a^	0.13 ± 0.04 ^fg^	1.54 ± 0.05 ^b^	-	0.36 ± 0.05 ^c^	1.44 ± 0.24 ^bc^	0.49 ± 0.12 ^d^	1.77 ± 0.04 ^a^	0.55 ± 0.45 ^d^
	Monoterpenoids Hydrocarbon			3.02 ± 0.07	1.20 ± 0.02	-	-	1.25 ± 0.03	16.84 ± 0.29	0.28 ± 0.05	1.90 ± 0.03	0.36 ± 0.13	1.81 ± 0.21	6.43 ± 0.03
	Monoterpenoids Alcohol			3.67 ± 0.04	3.70 ± 0.31	0.80 ± 0.07	1.87 ± 0.64	2.56 ± 0.07	8.20 ± 0.08	2.40 ± 0.23	2.42 ± 0.05	1.88 ± 0.87	5.59 ± 0.32	3.14 ± 0.08
	Sesquiterpenoid Hydrocarbon			59.81 ± 0.38	56.24 ± 0.74	59.37 ± 0.03	65.63 ± 0.35	54.29 ± 0.17	52.83 ± 0.47	60.73 ± 0.43	59.80 ± 0.14	63.64 ± 0.80	58.41 ± 0.64	60.49 ± 0.26
	Sesquiterpenoid Alcohol			17.97 ± 0.25	19.80 ± 0.15	14.71 ± 0.21	14.97 ± 0.53	22.45 ± 0.25	15.75 ± 0.24	16.05 ± 0.08	16.07 ± 0.08	17.79 ± 0.34	20.49 ± 0.39	17.24 ± 0.29
	Other			5.77 ± 0.33	10.48 ± 0.25	17.68 ± 0.28	7.92 ± 0.09	10.91 ± 0.09	0.78 ± 0.09	13.50 ± 0.83	13.06 ± 0.23	13.76 ± 0.45	11.60 ± 0.03	11.60 ± 0.59
	Total			90.23 ± 2.51	91.42 ± 2.57	92.56 ± 2.58	90.40 ± 1.52	91.46 ± 2.48	94.40 ± 3.60	92.96 ± 1.89	93.25 ± 0.76	97.44 ± 1.53	97.90 ± 1.04	98.90 ± 0.30

- Compounds not detected. RI ^a^: Retention indices on Elite-5 column, experimentally determined using homologous series of C_8_-C_20_ n-alkanes. RI ^b^: Retention index taken from literature [31]. Identification methods: MS, comparison of mass spectra with NIST library; RI, comparison of retention index with those reported in literature. The mean having different letters in a row is significantly different according to Tukey’s HSD test at *p* < 0.05. * Each sample was analyzed in triplicate.

**Table 3:** Concentration (min and max) of top 10 constituents of *Magnolia champaca* leaf essential oil as identified by GC-MS.

Sl No.	Compounds	Concentration Range (%)	Accession No. with Highest %	Collection Site
1	β-Elemene	0.23–38.76	MCL9	Ganjam
2	γ-Muurolene	0.31–22.48	MCL48	Sundargarh
3	β-Caryophyllene	0.03–20.70	MCL16	Jajpur
4	Bicyclogermacrene	1.26–14.54	MCL42	Sundargarh
5	Methyl linoleate	1.14–10.60	MCL27	Khordha
6	Viridiflorene	0.16–11.02	MCL42	Sundargarh
7	n-Hexadecanol	1.43–9.47	MCL36	Nayagarh
8	Linalool	1.02–9.59	MCL17	Kendrapara
9	Germacrene B	0.06–9.78	MCL51	Sundargarh
10	α-Humulene	1.07–8.20	MCL1	Bhadrak

## Data Availability

The data presented in this study are available on request from the corresponding author.

## Sample Availability

Samples of the compounds are not available from the authors.
